# Preservation-induced distortions in aquatic protists: mechanistic biases, quantitative uncertainties and ecological consequences

**DOI:** 10.1093/plankt/fbag038

**Published:** 2026-05-29

**Authors:** Albert Calbet

**Affiliations:** Institut de Ciències del Mar, CSIC (Consejo Superior de Investigaciones Científicas), Department of Marine Biology and Oceanography, Passeig Marítim de la Barceloneta 37-49, 08003, Barcelona, Spain

**Keywords:** fixation, microzooplankton, microalgae, Lugol, glutaraldehyde

## Abstract

Aquatic protist quantification underpins our understanding of microbial food webs, biogeochemical cycling and ecosystem functioning. Yet, most estimates of abundance, biomass, size structure and community composition still rely on preserved samples, even though fixation alters the organisms it is meant to preserve. Here, I synthesize experimental, methodological and comparative studies spanning several decades to show that fixation is not neutral, but a systematic, taxon-dependent source of distortion. Across common fixatives, including Lugol’s iodine, formaldehyde, protargol and glutaraldehyde, cell volume may shrink by >80% or swell by 30–55%, depending on taxon and protocol. Abundance estimates are likewise biased: aldehyde-preserved samples often yield ciliate counts 10–50% lower than Lugol-preserved samples, with even greater losses in fragile naked taxa, while some flagellate staining workflows recover only 20–30% of live abundance. These effects are further modified by storage time, handling and analytical method, generating error cascades that affect biomass estimates, trophic interpretation and carbon flux calculations. Because these artifacts vary across taxa and workflows, universal correction factors are not appropriate. Instead, fixation acts as a selective ecological filter, reshaping plankton communities before observation.

## INTRODUCTION

Aquatic protists form the functional core of pelagic ecosystems, mediating energy transfer between primary producers and higher trophic levels, recycling nutrients and regulating carbon flux across marine and freshwater environments (Azam *et al.,* 1983; Fenchel, 1988; Sherr and Sherr, 2002). Quantifying their abundance, biomass and community composition remains a central objective in plankton ecology, underpinning our understanding of microbial food webs, trophic coupling and biogeochemical cycling. Despite advances in imaging, flow cytometry and molecular approaches, most ecological studies continue to rely on preserved samples, particularly for microscopy-based enumeration and size estimation (Utermöhl, 1958; Hasle, 1978; Throndsen, 1978; Vaulot *et al.,* 1989). Direct microscopic inspection of preserved samples also has an important ecological advantage beyond enumeration and sizing: it can reveal recently ingested prey and thereby link protist identity to trophic function *in situ*. Such observations have documented bacterivory by pelagic ciliates and the ingestion of large diatom chains by gymnodinioid dinoflagellates and spirotrich ciliates, although prey visibility and food-vacuole integrity may themselves be affected by preservation artifacts (Sherr and Sherr, 1987; Sherr *et al.,* 2013).

This reliance reflects both the logistical constraints inherent to field sampling—particularly the temporal separation between collection and analysis—and the intrinsic fragility of protistan cells, which are highly susceptible to mechanical disruption and degradation during handling (Gifford, 1985; Sherr and Sherr, 1993; Stoecker *et al.,* 1994). However, the assumption that fixation preserves the ecological state of plankton communities has long been recognized as problematic. Protists are structurally delicate and especially vulnerable to disturbance during preservation (Sherr and Sherr, 1993), and numerous studies have documented substantial cell losses, morphological alterations and changes in biovolume following fixation (Bloem *et al.,* 1986; Choi and Stoecker, 1989; Sime-Ngando and Grolière, 1991; Leakey *et al.,* 1994; Modigh and Castaldo, 2005). These artifacts have direct consequences for ecological interpretation. Biomass estimates depend on cell volume, which is systematically altered by fixation, while abundance estimates are affected by selective cell loss and taxon-specific preservation efficiency (Montagnes *et al.,* 1994; Menden-Deuer *et al.,* 2001). Consequently, preserved samples do not represent a static snapshot of the living community but rather a transformed assemblage shaped by chemical and physical processes acting at the cellular level.

The magnitude of these transformations is substantial and highly variable across taxa and fixation methods. Experimental evidence shows that fixation can induce bidirectional changes in protist biovolume, ranging from strong shrinkage to moderate swelling, depending on taxon, preservative and analytical workflow (Chaput and Carrias, 2002). Abundance estimates are likewise sensitive to preservation method, but the strength of the bias depends on whether the comparison is made against live samples or against an alternative fixative. For ciliates, comparative studies consistently show higher counts in Lugol-preserved than in aldehyde-preserved samples, with differences commonly on the order of ~ 10–50% and sometimes larger in fragile naked forms (Leakey *et al.,* 1994; Stoecker *et al.,* 1994; Modigh and Castaldo, 2005). These contrasts indicate important fixative-specific differences in detectability and preservation efficiency, but they do not necessarily represent direct losses relative to the living community, because many estimates are based on comparisons among preservatives rather than live-versus-fixed samples. In contrast, heterotrophic flagellates show highly protocol-dependent responses: some unbuffered aldehyde formulations preserve counts reasonably well over short periods, whereas buffered formulations or inappropriate handling can cause severe apparent losses or even complete disappearance of cells (Bloem *et al.,* 1986). More generally, heterotrophic protists are highly susceptible to handling and preservation artifacts, and partial lysis or loss during fixation is widely recognized as a major source of underestimation in abundance estimates (Sherr and Sherr, 1993).

Importantly, fixation effects are not limited to immediate chemical interactions but evolve over time. Storage duration is therefore a critical determinant of sample integrity. This issue is particularly relevant for long oceanographic expeditions, where preserved samples may remain onboard for weeks before laboratory processing. In such cases, storage time becomes part of the analytical protocol itself, rather than a neutral delay between sampling and counting. Early work on freshwater ciliates showed progressive declines during storage, with mean losses of 7.2% after 3 months, 16.4% after 6 months, and 30.7% after 9 months across fixatives, and with Lugol performing particularly poorly for long-term ciliate preservation (Sime-Ngando and Grolière, 1991). More recent studies confirm that phytoplankton and protist abundances can also decline substantially over time, with rapid reductions in pico- and nanoplankton occurring within days and continued losses in larger microplankton over longer periods (Mukherjee *et al.,* 2014; Nogueira *et al.,* 2023). These temporal dynamics introduce an additional source of variability, complicating comparisons across studies and potentially biasing long-term datasets.

The problem is further compounded by the diversity of fixation methods and analytical approaches used in plankton research. Lugol’s iodine, formaldehyde, glutaraldehyde and their combinations each introduce distinct artifacts, while techniques such as flow cytometry impose additional constraints on preservation due to the need to maintain fluorescence signals (Vaulot *et al.,* 1989; Liu *et al.,* 2022). Methods optimized for one objective often compromise others, creating unavoidable trade-offs between morphological integrity, abundance accuracy and biochemical preservation. As a result, no single fixation protocol can simultaneously preserve all relevant aspects of plankton communities, and methodological choices inevitably influence the observed ecological patterns.

Despite decades of research documenting these effects, fixation-induced biases remain insufficiently integrated into ecological analyses. Correction factors are applied inconsistently, and key methodological details—including fixative type, concentration, storage time and handling procedures—are often incompletely reported, limiting the comparability of datasets (Jakobsen and Carstensen, 2011). More fundamentally, fixation is typically treated as a neutral preparatory step rather than as an active process that alters the system under investigation.

Here, I argue that fixation should be understood as a selective ecological filter operating along multiple axes—taxonomic, morphological and temporal—that transforms plankton communities prior to observation. By synthesizing experimental and methodological evidence, I propose a quantitative framework linking fixation mechanisms to cellular responses and ecological consequences. This framework highlights the cascading nature of preservation artifacts, from molecular and structural changes at the cellular level to biases in abundance, biomass and trophic interactions at the ecosystem scale. In this review, I use the term protists in an operational plankton-ecology sense, focusing primarily on phagotrophic and mixotrophic taxa involved in microbial food-web processes, especially ciliates, dinoflagellates and heterotrophic or mixotrophic nanoflagellates. Strictly autotrophic microalgae are only considered where preservation effects on abundance, size or biomass provide relevant methodological comparisons. The objectives of this review are therefore threefold: (i) to quantify the magnitude and variability of fixation-induced biases across taxa and methods, (ii) to elucidate the mechanisms underlying these biases and (iii) to propose pathways toward more accurate and comparable plankton measurements through bias-aware protocols and correction strategies.

## MECHANISMS OF FIXATION: CHEMICAL AND PHYSICAL DRIVERS OF CELLULAR TRANSFORMATION

Fixation alters protistan cells through a combination of chemical reactions, osmotic disequilibria and mechanical stresses that operate simultaneously and whose relative importance depends on both fixative chemistry and organismal traits. These processes do not simply “preserve” cellular structure but actively transform it, producing systematic and often predictable deviations from the living state. A mechanistic understanding of these transformations is essential, because it provides the causal link between fixation protocols and the quantitative biases described in subsequent sections.

Aldehyde-based fixatives, primarily formaldehyde and glutaraldehyde, act through covalent cross-linking of proteins and other macromolecules, thereby arresting metabolic activity and stabilizing cellular architecture. Formaldehyde reacts with amino groups to form methylene bridge cross-links between adjacent residues, while glutaraldehyde, as a bifunctional aldehyde, produces more extensive intermolecular cross-linking networks. These stronger cross-links increase structural rigidity and are often associated with enhanced cytoplasmic contraction and reduced cellular volume (Kiernan, 2000), but responses are strongly taxon-specific and can also include little change or swelling (Choi and Stoecker, 1989; Chaput and Carrias, 2002). The magnitude of shrinkage is not constant but depends on fixative concentration, exposure time and cellular composition, with smaller and more weakly structured cells generally exhibiting stronger responses (Bloem *et al.,* 1986; Modigh and Castaldo, 2005).

In contrast, iodine-based fixatives such as Lugol’s solution act through a combination of chemical interactions and osmotic effects rather than covalent cross-linking. Iodine reacts with cellular constituents, including proteins and polysaccharides, while potassium iodide enhances iodine solubility and penetration into cells. In addition, the degree of acidification influences preservation efficiency and cell morphology, introducing further variability in fixation outcomes (Throndsen, 1978; Sherr and Sherr, 1993). Acid Lugol, the most widely used formulation in plankton studies, lowers pH and promotes rapid fixation but also enhances dehydration and structural collapse in many taxa. Neutral (non-acidified) Lugol, by maintaining a higher pH, may reduce some of these effects, although available comparisons suggest strongly taxon-specific responses rather than a consistent across-the-board advantage (Williams *et al.,* 2016). It is also important to distinguish among Lugol formulations. Home-made and commercial products do not necessarily behave equivalently, and at least one comparison reported drastic community damage with a commercial preparation, underscoring that formulation details can strongly affect preservation outcome (Calbet *et al.,* 2011; Williams *et al.,* 2016). Because Lugol’s solution does not stabilize cells through covalent cross-linking, its effects depend strongly on iodine concentration, acidity and the osmotic contrast between fixative and sample. Its practical advantages—improved contrast, enhanced sedimentation and easier recognition of many taxa—can therefore coexist with iodine-induced staining, altered optical properties, aggregation and collapse of weakly supported cells. Lugol should consequently be regarded as a useful but chemically active preservative, not as a benign default.

Osmotic stress is a central but often underemphasized component of fixation. The introduction of fixatives alters the ionic and chemical environment surrounding cells, driving water flux across membranes and leading to rapid deformation. In naked ciliates and flagellates, which are essentially bounded by flexible membranes, these osmotic shocks can result in transient swelling followed by collapse or fragmentation (Sherr and Sherr, 1993). Observations of freshly fixed samples frequently report distorted morphologies, including inflated or irregular cell shapes, which subsequently stabilize as cross-linking or chemical equilibration proceeds. The direction and magnitude of these changes depend on both the external solution and the internal composition of the cell, explaining the high variability observed across taxa.

Mechanical processes associated with sample handling interact strongly with these chemical and osmotic effects. Filtration, centrifugation and mixing impose shear forces that can damage or selectively remove cells, particularly when fixation has altered their mechanical properties. For example, heterotrophic nanoflagellates subjected to filtration at moderate vacuum pressures exhibit losses of 15–20%, with higher losses under increased pressure or clogging conditions (Bloem *et al.,* 1986). Centrifugation produces comparable effects even at relatively low speeds, indicating that mechanical stress alone can substantially bias abundance estimates. Mechanical stress is especially important because fixation changes the material properties of cells before they are filtered, concentrated, mixed or settled. Cross-linked cells may become rigid and brittle, whereas Lugol-fixed cells may remain deformable, swollen or partially collapsed. The same filtration or centrifugation step can therefore generate different losses depending on the preceding fixative.

Taken together, these mechanisms—chemical cross-linking, osmotic imbalance, mechanical stress and temporal degradation—operate in concert to transform plankton cells in ways that are systematic but highly context-dependent. The resulting biases are not random artifacts but the predictable outcome of interactions between fixative chemistry and organismal traits. This mechanistic framework explains why different fixatives yield different estimates of abundance and size, why these differences vary across taxa and why they evolve over time. It also underscores a central point of this synthesis: fixation is not a neutral preservation step but an active process that reshapes the biological system under observation.

## QUANTITATIVE BIASES IN ABUNDANCE, BIOVOLUME AND SIZE STRUCTURE

**Fig. 1:**
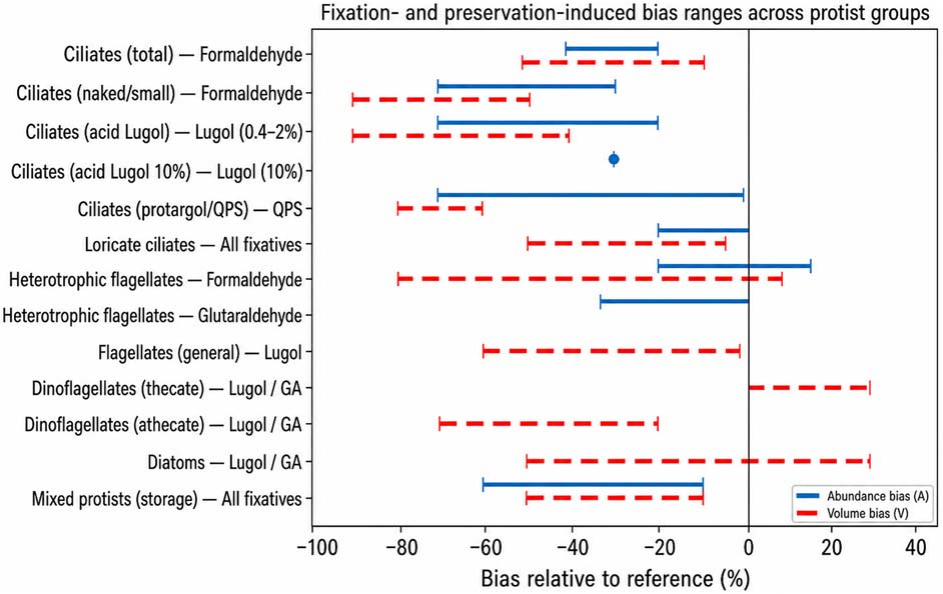
Fixation- and preservation-induced bias ranges in abundance and cell volume across major protist groups. Solid blue lines indicate reported ranges of abundance bias, and dashed red lines indicate reported ranges of volume bias, expressed as percentage change relative to the reference used in each study (live samples, initial counts, post-fixation baseline or an alternative fixative). Negative values denote lower apparent abundance, cell shrinkage or reduced detectability after preservation, whereas positive values denote higher apparent abundance or swelling. Values shown are synthesized from the studies reviewed here and represent approximate empirical envelopes rather than formal confidence intervals. Metrics are omitted where the available evidence did not allow a defensible constraint. The figure is intended to provide a comparative overview of the magnitude, direction and taxon-specific variability of preservation artifacts relevant to quantitative protist ecology.

The mechanistic processes induced by fixation translate into substantial and structured quantitative biases in the core descriptors of plankton communities, namely abundance, cell volume and size distributions (Fig. 1; Table 1). These biases are not minor methodological uncertainties but often reach magnitudes large enough to confound, or even exceed, the ecological differences being compared. Importantly, they are neither random nor uniform; rather, they reflect the interaction between fixative chemistry, organismal traits and methodological workflows, producing systematic distortions that propagate through ecological analyses.

**Table 1:** Summary of fixation- and preservation-induced biases across protist groups, with approximate bias ranges and suggested correction factors synthesized from the studies analyzed here. Abundance (A) bias is expressed relative to the reference indicated in the table and reported as the percentage change in preserved samples compared with live samples, initial counts, post-fixation baseline or an alternative fixative, depending on study design. Volume (V) bias is expressed relative to the corresponding reference indicated in the table. Negative values indicate lower abundance, shrinkage or reduced detectability after preservation; positive values indicate higher apparent abundance or swelling. Suggested correction factors are approximate inverse multipliers intended only as first-order guidance within the taxonomic and methodological bounds of the cited studies; they should not be applied uncritically across systems, taxa or protocols. Where the underlying evidence is indirect, mixed or taxon-specific, correction ranges should be treated cautiously

Target group	Fixative	Abundance bias (%)	Reference type (A)	Volume bias (%)	Reference type (V)	Suggested correction (A/V)	Notes	Key references
Ciliates (total)	Formaldehyde	−20 to −40	Indirect (vs Lugol)	−10 to −50	Live (partial)	×1.3–1.7/×1.1–2.0	Abundance derived mainly from Lugol comparisons; volume partly constrained by live-based estimates	Stoecker *et al.,* 1994; Leakey *et al.,* 1994; Modigh and Castaldo, 2005
Ciliates (naked/small)	Formaldehyde	−30 to −70	Live	−50 to −90	Live	×1.5–3.3/×2–10	Strong collapse and loss of fragile taxa	Pfister *et al.,* 1999; Chaput and Carrias, 2002
Ciliates (acid Lugol)	Lugol (0.4–2%)	−20 to −70	Mixed (live + indirect)	−40 to −90	Live	×1.3–3.3/×1.7–10	Includes direct live comparisons for some cases but also strong size-redistribution effects in mixed communities	Zarauz and Irigoien, 2008; Yang *et al.,* 2017
Ciliates (acid Lugol 10%)	Lugol (10%)	−30	Live	Not constrained	—	×1.4/—	Species-specific estimate for *Strombidium sulcatum*; should not be generalized to all naked ciliates	Broglio *et al.,* 2004
Ciliates (protargol/QPS)	QPS	−1 to −70	Live	−60 to −80	Live	×1.0–3.3/×2.5–5	Strongly taxon-dependent over- and underestimation	Pfister *et al.,* 1999
Loricate ciliates	All fixatives	0 to −20	Indirect + partial live	−5 to −50	Mixed	×1.0–1.2/×1.1–2.0	Structurally more resistant taxa	Leakey *et al.,* 1994; Pfister *et al.,* 1999
Heterotrophic flagellates	Formaldehyde	−20 to +15	Live	−80 to +8	Live	×0.9–1.3/×1.0–5.0	Includes taxon-specific abundance responses and both swelling and shrinkage in cell volume	Sonntag *et al.,* 2000; Chaput and Carrias, 2002
Heterotrophic flagellates	Glutaraldehyde	0 to −33	Mixed (live + post-fixation baseline)	Not constrained	—	×1.0–1.5/—	Stable in some HNAN counts, but buffered formulations can cause substantial losses; major fluorescence artifacts concern PNAN more than HNAN	Bloem *et al.,* 1986
Flagellates (general)	Lugol	Not constrained	—	−60 to −2	Live	—/×1.0–2.5	No robust abundance-vs-live constraint across taxa	Chaput and Carrias, 2002
Dinoflagellates (thecate)	Lugol/GA	Not constrained	—	0 to +30	Live	—/×0.8–1.0	Abundance response not defensibly constrained from the cited studies; Measured swelling is common in some preserved thecate taxa	Menden-Deuer *et al.,* 2001; Yang *et al.,* 2017
Dinoflagellates (athecate)	Lugol/GA	Not constrained	—	−20 to −70	Live	—/×1.2–3.3	Fragile taxa; shrinkage and rupture dominate	Menden-Deuer *et al.,* 2001; Yang *et al.,* 2017
Diatoms	Lugol/GA	Not constrained	—	−50 to +30	Live	—/×0.8–2.0	No consistent response across taxa	Menden-Deuer *et al.,* 2001; Nogueira *et al.,* 2023
Mixed protists (storage)	All fixatives	−10 to −60	Time-series (not live baseline)	−10 to −50	Mixed	—/—	Time-dependent decay during storage; values are not direct live-based correction ranges and should be interpreted only as indicative loss envelopes	Sime-Ngando and Grolière, 1991; Nogueira *et al.,* 2023

*Footnote.* Bias ranges are synthesized from the studies reviewed here and represent approximate envelopes rather than formal meta-analytic confidence intervals. Reference type (A), abundance reference; reference type (V), volume reference. Suggested correction (A/V) indicates approximate inverse multiplicative factors for translating preserved estimates toward live-equivalent abundance or volume. QPS, quantitative protargol stain; GA, glutaraldehyde. “Mixed” indicates that both direct live comparisons and indirect or derived comparisons contributed to the synthesis. “Indirect” indicates that the estimate is based primarily on comparisons among preservatives rather than direct live-preserved contrasts. “Not constrained” indicates that the available studies did not support a defensible general range for that variable across the target group. “Live (partial)” indicates that only part of the evidence base used direct live comparisons. Because responses are strongly taxon-, protocol-, concentration- and storage-dependent, these ranges and correction factors should be interpreted as pragmatic guidance rather than universal corrections.

Abundance estimates are particularly sensitive to fixation (Table 1; Supplementary Table 1), as they integrate multiple processes including cell lysis, deformation, aggregation and changes in detectability. Comparative studies consistently show that different fixatives yield markedly different counts for the same community. In marine ciliates, for example, comparative studies generally show higher abundances in Lugol than in aldehyde-preserved samples, with indirect differences commonly on the order of ~ 10–50%, depending on taxon and protocol (Leakey *et al.,* 1994; Stoecker *et al.,* 1994; Modigh and Castaldo, 2005). These discrepancies arise primarily from the greater susceptibility of fragile taxa to aldehyde-induced damage and subsequent loss. Direct comparisons with live counts further highlight the magnitude of the problem, but they also show that the bias is method- and taxon-specific: Broglio *et al.* (2004) reported a 30% loss for *Strombidium sulcatum* in 10% acid Lugol, whereas Pfister *et al.* (1999) showed that QPS can recover total ciliate abundance reasonably well overall while strongly underestimating fragile taxa and reducing some measured flagellate abundances to roughly one-third of live values. In extreme cases, inappropriate buffering of aldehydes can result in near-complete loss of flagellates within hours (Bloem *et al.,* 1986), illustrating the sensitivity of these organisms to subtle chemical conditions.

Biases in cell volume are equally pronounced and have direct implications for biomass estimation (Table 1). Measurements across a wide range of protists demonstrate that fixation can induce volume changes spanning from substantial shrinkage to moderate swelling, with reported ranges of −64% to +59% depending on the fixative and taxon (Chaput and Carrias, 2002). Aldehyde fixatives often induce shrinkage, but responses are not uniform and may include little change or even swelling, especially when species with contrasting responses are pooled together (Choi and Stoecker, 1989; Sonntag *et al.,* 2000; Chaput and Carrias, 2002; Yang *et al.,* 2017; Supplementary Table 1). In contrast, staining-based approaches can induce far more pronounced distortions: for example, after QPS, some ciliates may retain <20% of their original live volume, corresponding to shrinkage exceeding 80% (Pfister *et al.,* 1999). These results highlight the potential for severe, method-dependent biases in cell volume estimates.

The implications of these volume changes extend beyond simple size measurements. Biomass estimates are typically derived from cell volume using empirical carbon–volume relationships, often expressed as power laws (Menden-Deuer and Lessard, 2000). Because these relationships are nonlinear, errors in volume estimation are amplified when converted to carbon biomass. For example, a 50% reduction in cell volume can lead to disproportionately large underestimation of biomass, particularly for larger cells that contribute significantly to total carbon pools. Moreover, because shrinkage varies across taxa, the resulting biomass distribution becomes skewed, altering the relative contribution of different functional groups.

The combined effects of abundance and volume biases lead to systematic distortion of size structure within plankton communities. Size spectra, which describe the distribution of biomass or abundance across size classes, are widely used to infer ecological processes such as trophic transfer and energy flow. Fixation-induced shrinkage compresses size distributions, while selective loss of larger or more fragile cells shifts the spectrum toward smaller size classes. Empirical comparisons between live and preserved samples analyzed with imaging systems such as FlowCAM demonstrate that fixation can significantly alter both total particle counts and the shape of size distributions, even when general patterns appear similar (Jakobsen and Carstensen, 2011). These distortions are particularly important because size-based metrics are often used as proxies for ecosystem state.

Temporal dynamics further complicate these quantitative biases. Abundance and volume do not remain constant after fixation but evolve during storage, with rates that depend on taxon, fixative and environmental conditions. Experimental studies show that phytoplankton abundance declines progressively over time, with significant reductions occurring within days for pico- and nanoplankton and continuing over weeks to months for larger cells (Nogueira *et al.,* 2023). In some cases, cumulative losses exceeding 50% have been observed during prolonged storage (Sime-Ngando and Grolière, 1991). These changes are not uniform across taxa, leading to additional shifts in community composition and size structure that are independent of ecological processes.

Taken together, the quantitative evidence demonstrates that fixation-induced biases in abundance, volume and size structure are large, systematic and context-dependent. Their magnitude often exceeds the natural variability observed in plankton communities, meaning that methodological effects can dominate ecological signals if not properly accounted for. Moreover, the interaction between different sources of bias—chemical, mechanical and temporal—produces compound effects that cannot be captured by simple correction factors. Instead, these biases must be understood as the quantitative expression of the mechanistic processes described earlier, linking fixation chemistry to ecological interpretation.

## TAXON-SPECIFIC RESPONSES TO FIXATION: DIFFERENTIAL VULNERABILITY AND BIAS STRUCTURE

The quantitative biases induced by fixation are fundamentally structured by taxonomic identity, reflecting differences in cell architecture, biochemical composition and mechanical resilience among protistan groups (Fig. 1; Table 1). These differences determine not only the magnitude of shrinkage or cell loss but also the direction and variability of responses to specific fixatives. As a consequence, preservation therefore introduces a directional taxonomic bias, rather than a uniform loss across the community, in a predictable yet complex manner, favoring taxa that are structurally robust or chemically resistant while disproportionately removing or distorting fragile organisms. This taxon-specificity is central to understanding why correction factors cannot be generalized across communities and why preserved assemblages often diverge qualitatively from their living counterparts.

Ciliates, particularly aloricate forms, represent one of the most sensitive groups to fixation-induced artifacts. Their cells lack rigid external structures and consist largely of a highly hydrated cytoplasm bounded by a flexible membrane, making them especially vulnerable to osmotic and mechanical stress. Numerous studies have documented substantial losses and shrinkage in ciliates following fixation. Aldehyde-based methods often underestimate ciliate abundance relative to Lugol, commonly by ~11–48% in indirect fixative comparisons for total or aloricate assemblages, with larger losses in fragile naked forms when assessed against live material or staining-based methods (Leakey *et al.,* 1994; Stoecker *et al.,* 1994; Pfister *et al.,* 1999; Modigh and Castaldo, 2005). Ciliate size responses are also strongly taxon-specific, ranging from shrinkage in many fragile forms to swelling in some taxa (Choi and Stoecker, 1989; Chaput and Carrias, 2002), and reaching extreme values under protargol-based workflows, where some taxa are reduced to <20% of live volume (Pfister *et al.,* 1999). Importantly, these responses are not uniform within the group. Aloricate oligotrichs and choreotrichs are particularly susceptible to deformation and lysis, whereas loricate forms, protected by a rigid lorica, exhibit greater resistance and more stable morphology. This differential response leads to systematic shifts in ciliate community structure, with armored taxa becoming overrepresented in preserved samples.

Heterotrophic nanoflagellates and mixotrophic flagellates often exhibit even greater sensitivity to fixation than ciliates, largely because of their small size and limited structural reinforcement. Their responses, however, are exceptionally method-dependent. In cultured and field material, some unbuffered aldehyde protocols preserved nanoflagellate counts reasonably well over short periods, whereas buffered formulations caused strong declines or even complete disappearance of cells (Bloem *et al.,* 1986). In addition, some apparent losses reflect changes in detectability rather than true disappearance, as fluorescence decay can shift phototrophic cells into heterotrophic categories during storage (Bloem *et al.,* 1986). Live-versus-fixed comparisons also show that both abundance and cell volume responses vary sharply among species and methods, with some taxa showing little change and others exhibiting strong shrinkage or underestimation (Sonntag *et al.,* 2000; Chaput and Carrias, 2002). These results indicate that nanoflagellates are not uniformly poorly preserved; rather, they are exceptionally sensitive to the combined effects of fixative chemistry, buffering, storage and detection method.

Lugol-based fixatives also show strong taxon-specific variability. For ciliates they often yield higher counts than aldehydes, even though shrinkage, aggregation and distortion may remain substantial (Sherr and Sherr, 1993; Williams *et al.,* 2016). For flagellates, however, the available evidence does not support a single abundance correction across taxa, because responses span near-stability, shrinkage, swelling and major detectability problems depending on the species and workflow (Sonntag *et al.,* 2000; Chaput and Carrias, 2002). Given their central role in the microbial loop, this uneven preservation has significant implications for estimates of bacterial grazing and nutrient recycling.

Dinoflagellates display a broad spectrum of responses to fixation, reflecting their marked structural diversity. Thecate forms, protected by cellulose plates, are often more resistant to gross deformation than athecate forms, and some studies report little change or even moderate swelling in measured volume after preservation (Menden-Deuer *et al.,* 2001; Yang *et al.,* 2017). By contrast, athecate dinoflagellates are generally more fragile and more prone to shrinkage, deformation or rupture, although the magnitude of these effects remains strongly species- and protocol-dependent. Acid Lugol is widely used for dinoflagellate and ciliate enumeration because it improves visibility and sedimentation, but its effects are not uniformly benign: some taxa shrink, others remain relatively stable and prolonged storage can reduce abundance or alter appearance (Nogueira *et al.,* 2023). Aldehyde fixatives are often preferred for cytometric or fluorescence-based applications; yet, they can also modify morphology and complicate taxonomic identification by microscopy (Sherr and Sherr, 1993). Neutral Lugol formulations have been explored as alternatives, but available evidence suggests variability rather than a consistent across-the-board improvement (Hällfors *et al.,* 1979; Williams *et al.,* 2016).

Diatoms and other phytoplankton with rigid cell coverings are generally less affected by fixation in terms of external morphology, owing to the protective role of silica frustules or other structural components. As a result, their abundance estimates are often more robust than those of more fragile taxa over short storage periods. However, this apparent stability should not be overstated. Live-based size measurements show no single consistent response even within diatoms, with reported volume changes ranging from ~−33% to +28% across species (Menden-Deuer *et al.,* 2001). Moreover, long-term storage can still reduce the abundance of some phytoplankton groups substantially, especially among smaller cells, even when larger rigid forms appear comparatively stable (Nogueira *et al.,* 2023). Morphological preservation therefore does not necessarily imply analytical accuracy, because fixation can alter internal cellular contents, optical properties and sedimentation behavior, all of which influence detection, identification and quantification.

Across all taxa, the interaction between structural characteristics and fixative chemistry produces a consistent pattern of differential vulnerability. Naked, flexible cells are highly susceptible to both chemical and mechanical damage, whereas armored or structurally reinforced cells are more resistant. This pattern is further modulated by cell size, with smaller cells generally experiencing greater relative losses and shrinkage, and likely by physiological state, which influences cellular composition and mechanical properties. The result is a systematic reshaping of community composition during fixation, in which certain groups are selectively reduced or distorted while others are relatively preserved.

This taxonomic differential preservation has direct implications for ecological interpretation. Because different groups occupy distinct functional roles within plankton ecosystems, their differential preservation leads to biased representations of trophic structure and biogeochemical processes. For example, underrepresentation of heterotrophic flagellates and ciliates can lead to underestimation of grazing pressure and microbial loop activity, while the relative stability of some diatoms can exaggerate their apparent contribution to biomass and production. Such biases have direct implications for ecosystem-level interpretations. For instance, Dolan and McKeon (2005) questioned the global synthesis of microzooplankton grazing rates reported by Calbet and Landry (2004), arguing that dilution-based estimates may be systematically biased and potentially overestimated when evaluated against grazer abundances and plausible clearance rates. Their analysis showed that some inferred grazing rates were inconsistent with observed ciliate concentrations, implying unrealistically high per-cell feeding rates. However, this reasoning implicitly assumes that ciliate abundances are accurately quantified. Given that fixation and sample processing can lead to substantial losses or underestimation of ciliates, particularly fragile forms, part of the apparent mismatch between grazing rates and grazer abundance could, at least in part, reflect methodological undercounting rather than true ecological inconsistency. In response, Landry and Calbet (2005) emphasized that grazing estimates derived from dilution experiments reflect community-level processes and need not scale directly with any single grazer group, particularly given shifts in community composition and the role of non-ciliate grazers in open-ocean systems.

More broadly, this debate illustrates how biases in cell counts propagate into derived ecological rates: errors in estimating grazer abundance or prey availability can translate directly into misestimation of grazing pressure and carbon fluxes, ultimately affecting our understanding of trophic dynamics in planktonic ecosystems.

## FURTHER METHODOLOGICAL CAVEATS

Fixation-induced biases cannot be understood in isolation because they are embedded within a broader methodological continuum that includes sampling, handling, storage and observation. Each of these steps modifies the sample, and, critically, the effects are not additive but interactive. The outcome is a coupled system in which the final representation of the plankton community reflects the cumulative transformation imposed by the entire workflow. This coupling explains why similar communities processed with different protocols can yield markedly different quantitative and qualitative descriptions, and why correction approaches that target only fixation are often insufficient.

The first level of interaction occurs in handling prior to fixation, which can substantially affect initial abundance estimates of protozooplankton. Because many protists are highly fragile, mechanical stress during sampling and transfer can lead to cell breakage, deformation or loss before preservation occurs. For instance, Gifford (1985) demonstrated that even moderate turbulence and shear associated with routine handling can disrupt delicate protists, particularly ciliates, leading to significant losses of up to 60% of their original abundance. Subsequent methodological syntheses further emphasize that common procedures—such as draining sampling bottles, transferring water through tubing or pre-screening—can introduce substantial biases due to the mechanical sensitivity of protozooplankton (Gifford and Caron, 2000). As a result, pre-fixation handling can already reduce observed abundances and alter community composition, meaning that fixation artifacts act on a community that may have been substantially modified prior to preservation.

Once cells are chemically altered, their mechanical properties change, influencing how they respond to subsequent processing. Aldehyde fixation typically increases rigidity through cross-linking, which can make cells more resistant to deformation but also more brittle and susceptible to fragmentation under shear stress. In contrast, iodine-based fixatives such as Lugol’s solution may produce cells that are swollen or structurally weakened, increasing their vulnerability to rupture during manipulation. These altered mechanical states interact with common handling procedures such as filtration, centrifugation and mixing. Experimental work has shown that filtration alone can reduce heterotrophic nanoflagellate abundance by 15–20% under moderate vacuum pressure, with higher losses under more aggressive conditions (Bloem *et al.,* 1986). Centrifugation produces comparable reductions even at low speeds, indicating that mechanical stress is a significant source of bias independent of chemical fixation. When combined, these processes can produce compound losses that exceed those attributable to fixation alone.

Sedimentation-based methods, such as the Utermöhl technique, are generally considered less disruptive; yet, they are also influenced by fixation-induced changes in cell properties. Lugol, for example, increases cell density and enhances sedimentation efficiency, facilitating enumeration in inverted microscopy. However, it can also promote aggregation and alter cell morphology, potentially affecting both the distribution of cells on the counting surface and their identification (Utermöhl, 1958; Sherr and Sherr, 1993). The balance between improved detectability and structural distortion illustrates the trade-offs inherent in method selection, where gains in one aspect of measurement may be offset by losses in another.

The interaction between fixation and observational technique introduces a second level of coupling. Different analytical methods rely on different cellular properties—morphology, optical characteristics or biochemical signals—and fixation affects each of these in distinct ways. In brightfield microscopy, commonly used with Lugol-fixed samples, iodine staining enhances contrast and facilitates identification of many taxa. However, shrinkage and deformation can complicate size measurements and obscure diagnostic features, particularly in fragile groups such as ciliates and flagellates (Sherr and Sherr, 1993). In epifluorescence microscopy and flow cytometry, aldehyde fixation is often preferred because it preserves chlorophyll and nucleic acid fluorescence, enabling detection and classification based on optical signals. Yet, even in these applications, fixation can alter fluorescence intensity and light scattering properties, affecting detectability and potentially leading to underestimation of abundance or misclassification of cells (Vaulot *et al.,* 1989; Liu *et al.,* 2022).

Automated imaging systems provide a clear illustration of how fixation and detection interact. Comparisons between live and Lugol-fixed samples analyzed with FlowCAM show that fixation not only reduces cell counts but also alters size distributions and particle characteristics used for classification (Jakobsen and Carstensen, 2011). Changes in refractive index, shape and internal structure influence how cells are detected and segmented by image-analysis algorithms, leading to systematic differences in measured size spectra and community composition. These effects highlight that detection is not a neutral step but one that filters the already altered sample through method-specific criteria.

A further level of coupling arises when molecular and biochemical analyses are considered. Fixatives that preserve morphology or fluorescence may compromise nucleic acid integrity, while those optimized for molecular work may alter cellular structure. Aldehydes, for instance, form cross-links between nucleic acids and proteins that can inhibit deoxyribonucleic acid extraction and amplification, although protocols exist to partially reverse these effects. In contrast, preservation methods such as freezing or ethanol fixation are more suitable for molecular analyses but may introduce structural damage or loss of morphological information (Liu *et al.,* 2022). This incompatibility means that no single preservation method can simultaneously optimize all types of analysis, forcing researchers to make trade-offs or to process parallel samples using different protocols.

The cumulative effect of these interactions is a methodological cascade, in which each step modifies the outcome of the previous one. Fixation determines the initial state of the preserved cells, handling processes selectively remove or damage subsets of those cells, storage introduces time-dependent changes and observation techniques filter the remaining material based on specific detection criteria. The final dataset is therefore the product of a sequence of transformations rather than a direct measurement of the original community. Because each step depends on the others, changes in one component of the workflow can propagate through the system in nonlinear ways.

This coupled perspective also explains the variability observed across studies. Differences in fixative type, concentration, handling procedures, storage duration and analytical methods can produce divergent results even when sampling similar environments. For example, studies using acid Lugol and inverted microscopy may report higher ciliate abundances than those using aldehydes and fluorescence-based detection, not because of ecological differences but because of methodological filtering. Similarly, differences in filtration or centrifugation protocols can alter estimates of small flagellate abundance independently of fixation effects.

Recognizing methodological coupling has important implications for both experimental design and data interpretation. It highlights the need to consider the entire workflow when evaluating bias, rather than focusing on individual components in isolation. It also underscores the limitations of correction approaches that address only one aspect of the process, such as volume shrinkage, without accounting for concurrent abundance losses or detection biases. More broadly, it reinforces the central argument of this synthesis: the observed plankton community is a method-dependent construct, shaped by the interaction of biological properties and analytical procedures.

## PROPAGATION TO ECOLOGICAL METRICS AND CORRECTION FRAMEWORKS: FROM CELLULAR BIAS TO ECOSYSTEM INTERPRETATION

The biases introduced during fixation and subsequent methodological processing do not remain confined to primary measurements such as abundance and cell size, but propagate directly into the ecological metrics that underpin our understanding of plankton dynamics. Because these metrics—biomass, grazing rates, trophic transfer and carbon flux—are derived through a sequence of transformations, they integrate and often amplify underlying measurement errors. At the same time, the systematic nature of fixation-induced distortions provides an opportunity to quantify, constrain and partially correct these effects, provided that their multidimensional structure is explicitly recognized.

A central pathway of bias propagation is the conversion of cell volume into biomass. In plankton ecology, carbon biomass is typically estimated from cell volume using empirical scaling relationships (Menden-Deuer and Lessard, 2000). These relationships assume that measured volumes correspond to living cells; yet, fixation often induces substantial and taxon-specific changes in cell volume. In ciliates and flagellates, many protocols cause shrinkage on the order of 20–60%, but the full range is broader and bidirectional, extending from severe shrinkage exceeding 80% in some staining or preservation workflows to moderate swelling in certain taxa and fixatives (Pfister *et al.,* 1999; Sonntag *et al.,* 2000; Chaput and Carrias, 2002). Because volume–carbon relationships are nonlinear, these distortions are amplified during conversion, leading to systematic underestimation of biomass, particularly for larger cells that contribute disproportionately to total carbon pools. Consequently, even moderate shrinkage at the cellular level can translate into substantial errors at the community scale.

These effects are compounded by taxon-specific responses to fixation. Fragile organisms such as heterotrophic nanoflagellates and aloricate ciliates are disproportionately reduced in both abundance and volume (Bloem *et al.,* 1986; Stoecker *et al.,* 1994), whereas structurally robust taxa such as diatoms and thecate dinoflagellates are relatively preserved. This differential response redistributes apparent biomass toward resistant groups, altering the perceived balance between autotrophic and heterotrophic components of the community. Such distortions are particularly consequential in microbial food webs, where small protists play a central role in linking bacterial production to higher trophic levels (Azam *et al.,* 1983; Sherr and Sherr, 2002). Underestimation of these groups can therefore lead to systematic underestimation of microbial loop activity and associated carbon recycling.

Bias propagation extends directly into estimates of grazing and trophic interactions. Grazing rates are typically derived from prey abundance and size, combined with clearance or ingestion rates. Where prey or grazer abundances enter directly into biomass-normalized or clearance-based calculations, preservation-induced underestimation can propagate into grazing estimates. Similarly, shrinkage of predator cells can lead to underestimation of ingestion capacity, compounding errors in trophic flux calculations. Because these effects are taxon-specific, they alter the inferred strength and structure of trophic interactions, often weakening the apparent role of key grazers such as ciliates and flagellates.

Size-dependent processes introduce further complexity. Predator–prey interactions in plankton systems are strongly governed by size relationships, with feeding efficiency and selectivity determined by predator–prey size ratios (Hansen *et al.,* 1994). Fixation-induced shrinkage compresses these ratios, potentially altering inferred feeding dynamics. In addition, selective loss of larger or more fragile cells shifts size distributions toward smaller classes, distorting size spectra that are widely used to infer ecosystem structure and energy transfer. Empirical comparisons using imaging systems demonstrate that fixation alters both particle counts and size distributions, even when overall patterns appear similar (Jakobsen and Carstensen, 2011), highlighting the sensitivity of size-based metrics to methodological effects.

The cumulative outcome of these processes can be conceptualized as a cascade of bias propagation, in which initial distortions at the cellular level are transmitted and amplified through successive analytical steps. Fixation alters abundance and volume; these changes influence biomass estimates; biomass estimates affect calculations of grazing and trophic interactions; and these, in turn, inform models of carbon flux and ecosystem functioning. At each stage, errors introduced earlier in the chain are carried forward and often magnified, leading to potentially large discrepancies between observed and actual ecological processes.

Despite this complexity, the systematic nature of fixation-induced biases provides a basis for correction and interpretation. Traditional approaches have relied on empirical correction factors, such as multiplicative adjustments to account for average volume shrinkage, but these are inherently limited because they treat bias as a uniform scaling problem. In reality, fixation effects vary across taxa, size classes, fixative types and storage times (Choi and Stoecker, 1989; Sime-Ngando and Grolière, 1991), making universal correction factors inappropriate. Applying a single factor across mixed communities can therefore introduce additional bias rather than reducing it (Table 1).

A more robust framework requires recognizing that fixation imposes a multidimensional transformation on the community, in which observed values are a function of taxonomic composition, methodological conditions and time. Under this perspective, correction should not aim to recover a single “true” value, but rather to constrain the range of plausible values using taxon-specific and context-dependent adjustments. This can be achieved through a combination of approaches, including comparisons between live and fixed samples, parallel fixation experiments and time-series analyses of preserved material (Nogueira *et al.,* 2023). These approaches allow the construction of correction ranges rather than single values, providing a more realistic representation of uncertainty.

Incorporating uncertainty explicitly is critical because errors introduced at early stages of analysis are amplified through subsequent calculations. For example, variability in volume shrinkage propagates into biomass estimates and then into grazing and carbon flux calculations, often increasing in magnitude due to nonlinear scaling relationships. Representing these uncertainties as ranges or envelopes, rather than point estimates, allows a more accurate assessment of the robustness of ecological conclusions.

Ultimately, the most important implication is conceptual. Fixation-induced biases should not be viewed simply as errors to be corrected, but as structured transformations that reflect the interaction between biological properties and methodological processes. The observed community is therefore not a direct representation of the living system but a modified version shaped by fixation, handling, storage and detection. By characterizing this change and incorporating it into analysis, it becomes possible to reconcile preserved observations with ecological reality and to place quantitative constraints on uncertainty.

A simple implementation, although not always easy to conduct, would begin with paired live and preserved counts for the dominant taxa at the start of a study. For example, if Lugol-preserved aloricate ciliates are 30% lower than paired live counts and measured volumes are 40% lower, subsequent Lugol-based estimates should be reported as observed values together with a live-equivalent range based on the locally derived abundance and volume factors. In mixed assemblages, this procedure should be applied separately to ciliates, flagellates, dinoflagellates and rigid microalgae rather than using a single community-wide multiplier. Existing datasets lacking such paired comparisons should therefore be interpreted as method-conditioned estimates, especially when comparing fragile and resistant groups, cruises with different storage durations or studies using different preservatives.

In practical terms, bias-aware analysis should follow four principles. First, direct live-versus-fixed comparisons should be distinguished clearly from indirect contrasts among preservatives, because these two evidence types constrain different questions. Second, correction factors should be applied only within the taxonomic, concentration, storage and analytical bounds under which they were derived (Table 1). Third, abundance and volume corrections should be treated separately, because the two components often respond differently to the same workflow. Fourth, derived ecological quantities such as biomass, grazing and size spectra should be reported with uncertainty envelopes that explicitly incorporate preservation-related error rather than as single deterministic values. These steps will not eliminate preservation artifacts, but they will make their influence more transparent and more comparable across studies. Finally, it is evident we need better methods to preserve plankton samples; therefore, investing in this field should become a priority in future research.

## CONCLUSIONS

Several practical conclusions emerge from this synthesis:

First, fragile naked taxa, especially aloricate ciliates and heterotrophic nanoflagellates, are generally the most vulnerable to fixation, handling and storage artifacts, whereas loricate ciliates, thecate dinoflagellates and heavily silicified diatoms are usually more resistant.

Second, storage time should always be reported and, where possible, standardized. For long cruises or monitoring programs, delayed analysis can introduce systematic decreases in cell counts that may be mistaken for ecological differences.

Third, no fixative should be treated as universally optimal. Lugol’s solution remains useful for routine microscopy of many microplanktonic protists, especially when sedimentation and visual recognition are priorities, but it can distort cell size and damage fragile taxa. Aldehyde-based methods are often preferable for fluorescence-based analyses, but they may underestimate delicate ciliates and alter morphology.

Finally, the most robust strategy is not to search for a universal correction factor, but to combine consistent protocols, short and documented storage times, parallel live/fixed comparisons where feasible and taxon-specific uncertainty ranges when deriving biomass, grazing or carbon-flux estimates.

## Supplementary Material

fbag038_Supplementary_Table_1

## Data Availability

The dataset will be available in the DIGITAL.CSIC repository: https://doi.org/10.20350/digitalCSIC/18339.

## References

[ref1] Azam, F., Fenchel, T., Field, J. G., Gray, J. S., Meyer-Reil, L. A. and Thingstad, F. (1983) The ecological role of water-column microbes in the sea. Mar. Ecol. Prog. Ser., 10, 257–263. 10.3354/meps010257.

[ref2] Bloem, J., Bär-Gilissen, M. J. B. and Cappenberg, T. E. (1986) Fixation, counting, and manipulation of heterotrophic nanoflagellates. Appl. Environ. Microbiol., 52, 1266–1272. 10.1128/aem.52.6.1266-1272.1986. 16347232 PMC239220

[ref3] Broglio, E., Saiz, E., Calbet, A., Trepat, I. and Alcaraz, M. (2004) Trophic impact and prey selection by crustacean zooplankton on the microbial communities of an oligotrophic coastal area (NW Mediterranean Sea). Aquat. Microb. Ecol., 35, 65–78. 10.3354/ame035065.

[ref4] Calbet, A. and Landry, M. R. (2004) Phytoplankton growth, microzooplankton grazing, and carbon cycling in marine systems. Limnol. Oceanogr., 49, 51–57. 10.4319/lo.2004.49.1.0051.

[ref5] Calbet, A., Riisgaard, K., Saiz, E., Zamora, S., Stedmon, C. and Nielsen, T. G. (2011) Phytoplankton growth and microzooplankton grazing along a sub-Arctic fjord (Godthåbsfjord, West Greenland). Mar. Ecol. Prog. Ser., 442, 11–22. 10.3354/meps09343.

[ref6] Chaput, O. and Carrias, J.-F. (2002) Effects of commonly used fixatives on size parameters of freshwater planktonic protists. Arch. Hydrobiol., 155, 517–526. 10.1127/archiv-hydrobiol/155/2002/517.

[ref7] Choi, J. W. and Stoecker, D. K. (1989) Effects of fixation on cell volume of marine planktonic protozoa. Appl. Environ. Microbiol., 55, 1761–1765. 10.1128/aem.55.7.1761-1765.1989. 16347970 PMC202947

[ref9] Dolan, J. R. and McKeon, K. (2005) The reliability of grazing rate estimates from dilution experiments: have we over-estimated rates of organic carbon consumption by microzooplankton? Ocean Sci., 1, 1–7. 10.5194/os-1-1-2005.

[ref10] Fenchel, T. (1988) Marine plankton food chains. Annu. Rev. Ecol. Syst., 19, 19–38. 10.1146/annurev.es.19.110188.000315.

[ref11] Gifford, D. J. (1985) Laboratory culture of marine planktonic oligotrichs (Ciliophora, Oligotrichida). Mar. Ecol. Prog. Ser., 23, 257–267. 10.3354/meps023257.

[ref12] Gifford, D. J. and Caron, D. A. (2000) Sampling, preservation, enumeration and biomass of marine protozooplankton. In Harris, R. P., Wiebe, P. H., Lenz, J., Skjoldal, H. R. and Huntley, M. (eds.), ICES Zooplankton Methodology Manual, Academic Press, San Diego, pp. 193–221.

[ref13] Hällfors, G., Melvasalo, T., Niemi, A. and Viljamaa, H. (1979) Effect of different fixatives and preservatives on phytoplankton counts. Water Res Inst, 34, 25–34.

[ref14] Hansen, B., Bjørnsen, P. K. and Hansen, P. J. (1994) The size ratio between planktonic predators and their prey. Limnol. Oceanogr., 39, 395–403. 10.4319/lo.1994.39.2.0395.

[ref15] Hasle, G. R. (1978) The inverted-microscope method. In Sournia, A. (ed.), Phytoplankton Manual, UNESCO, Paris, pp. 88–96.

[ref16] Jakobsen, H. H. and Carstensen, J. (2011) FlowCAM: sizing cells and understanding the impact of size distributions on biovolume of planktonic community structure. Aquat. Microb. Ecol., 65, 75–87. 10.3354/ame01539.

[ref17] James, M. R. (1991) Sampling and preservation methods for the quantitative enumeration of microzooplankton. New Zealand J Mar Freshw Res, 25, 305–310. 10.1080/00288330.1991.9516483.

[ref18] Kiernan, J. A. (2000) Formaldehyde, formalin, paraformaldehyde and glutaraldehyde: what they are and what they do. Microscopy Today, 8, 8–13. 10.1017/S1551929500057060.

[ref19] Landry, M. R. and Calbet, A. (2005) Reality checks on microbial food web interactions in dilution experiments: responses to the comments of Dolan and McKeon. Ocean Sci., 1, 39–44. 10.5194/os-1-39-2005.

[ref20] Leakey, R. J. G., Burkill, P. H. and Sleigh, M. A. (1994) A comparison of fixatives for the estimation of abundance and biovolume of planktonic ciliate populations. J. Plankton Res., 16, 375–389. 10.1093/plankt/16.4.375.

[ref21] Liu, C., Lei, J., Zhang, M., Wu, F., Ren, M., Yang, J., Wu, Q. and Shi, X. (2022) Optimization of preservation methods provides insights into photosynthetic picoeukaryotes in lakes. Microbiol. Spectrum, 10, e02557–e02521. 10.1128/spectrum.02557-21. PMC9241741 35546573

[ref22] Menden-Deuer, S. and Lessard, E. J. (2000) Carbon to volume relationships for dinoflagellates, diatoms, and other protist plankton. Limnol. Oceanogr., 45, 569–579. 10.4319/lo.2000.45.3.0569.

[ref23] Menden-Deuer, S., Lessard, E. J. and Satterberg, J. (2001) Effect of preservation on dinoflagellate and diatom cell volume and consequences for carbon biomass predictions. Mar. Ecol. Prog. Ser., 222, 41–50. 10.3354/meps222041.

[ref24] Modigh, M. and Castaldo, S. (2005) Effects of fixatives on ciliates as related to cell size. J. Plankton Res., 27, 845–849. 10.1093/plankt/fbi053.

[ref25] Montagnes, J. S. D., Berges, J. A., Harrison, P. J. and Taylor, F. J. R. (1994) Estimating carbon, nitrogen, protein, and chlorophyll *a* from volume in marine phytoplankton. Limnol. Oceanogr., 39, 1044–1060. 10.4319/lo.1994.39.5.1044.

[ref26] Mukherjee, A., Das, S., Bhattacharya, T., De, M., Maiti, T. K. and De, T. K. (2014) Optimization of phytoplankton preservative concentrations to reduce damage during long-term storage. Biopreserv. Biobank., 12, 139–147. 10.1089/bio.2013.0074. 24749881

[ref27] Nogueira, P., Barbosa, A. B. and Domingues, R. B. (2023) Impacts of sample storage time on estimates of phytoplankton abundance: how long is too long? J. Plankton Res., 45, 794–802. 10.1093/plankt/fbad041.

[ref28] Pfister, G., Sonntag, B. and Posch, T. (1999) Comparison of a direct live count and an improved quantitative protargol stain (QPS) in determining abundance and cell volumes of pelagic freshwater protozoa. Aquat. Microb. Ecol., 18, 95–103. 10.3354/ame018095.

[ref29] Sherr, E. B. and Sherr, B. F. (1987) High rates of consumption of bacteria by pelagic ciliates. Nature, 325, 710–711. 10.1038/325710a0.

[ref30] Sherr, E. B. and Sherr, B. F. (1993) Preservation and storage of samples for enumeration of heterotrophic protists. In Kemp, P. F., Sherr, B. F., Sherr, E. B. and Cole, J. J. (eds.), Handbook of Methods in Aquatic Microbial Ecology, Lewis Publishers, Boca Raton, FL, pp. 207–212.

[ref31] Sherr, E. B. and Sherr, B. F. (2002) Significance of predation by protists in aquatic microbial food webs. Antonie Van Leeuwenhoek., 81, 293–308. 10.1023/A:1020591307260. 12448728

[ref32] Sherr, E. B., Sherr, B. F. and Ross, C. (2013) Microzooplankton grazing impact in the Bering Sea during spring sea ice conditions. Deep-Sea Res II, 94, 57–67. 10.1016/j.dsr2.2013.03.019.

[ref33] Sime-Ngando, T. and Grolière, C. A. (1991) Effets quantitatifs des fixateurs sur la conservation des ciliés planctoniques d’eau douce. Arch. Protistenkd., 140, 109–120. 10.1016/S0003-9365(11)80179-X.

[ref34] Sonntag, B., Posch, T. and Psenner, R. (2000) Comparison of three methods for determining flagellate abundance, cell size, and biovolume in cultures and natural freshwater samples. Arch. Hydrobiol., 149, 337–351. 10.1127/archiv-hydrobiol/149/2000/337.

[ref35] Stoecker, D. K., Gifford, D. J. and Putt, M. (1994) Preservation of marine planktonic ciliates: losses and cell shrinkage during fixation. Mar. Ecol. Prog. Ser., 110, 293–299. 10.3354/meps110293.

[ref36] Throndsen, J. (1978) Preservation and storage. In Sournia, A. (ed.), Phytoplankton Manual, UNESCO, Paris, pp. 69–74.

[ref37] Utermöhl, H. (1958) Zur Vervollkommnung der quantitativen phytoplankton-Methodik. Mitt Int Ver Theor Angew Limnol, 9, 1–38. 10.1080/05384680.1958.11904091.

[ref38] Vaulot, D., Courties, C. and Partensky, F. (1989) A simple method to preserve oceanic phytoplankton for flow cytometric analyses. Cytometry, 10, 629–635. 10.1002/cyto.990100519. 2505987

[ref39] Williams, O. J., Beckett, R. E. and Maxwell, D. L. (2016) Marine phytoplankton preservation with Lugol’s: a comparison of solutions. J. Appl. Phycol., 28, 1705–1712. 10.1007/s10811-015-0704-4.

[ref40] Yang, Y., Sun, X. and Zhao, Y. (2017) Effects of Lugol’s iodine solution and formalin on cell volume of three bloom-forming dinoflagellates. Chin. J. Oceanol. Limnol., 35, 858–866. 10.1007/s00343-017-5378-0.

[ref41] Zarauz, L. and Irigoien, X. (2008) Effects of Lugol’s fixation on the size structure of natural nano-microplankton samples, analysed by means of an automatic counting method. J. Plankton Res., 30, 1297–1303. 10.1093/plankt/fbn084.

