# Supplementary material for: Preservation-induced distortions in aquatic protists: mechanistic biases, quantitative uncertainties and ecological consequences
Source: J Plankton Res. 2026 May 29;48(3):fbag038. doi: 10.1093/plankt/fbag038 (PMC13220805; doi:10.1093/plankt/fbag038)
Supplement: fbag038_Supplementary_Table_1 [file fbag038_supplementary_table_1.docx]

**Supplementary Table 1.** Published effects of fixatives, preservatives, handling and storage on the abundance, detectability, and size or volume estimates of aquatic protists and related microbial eukaryotes. Abundance bias is expressed relative to the reference indicated in the table, including live samples, initial counts, post-fixation baseline, or an alternative fixative. Size or volume bias is likewise expressed relative to the stated reference. Positive values indicate higher abundance or larger measured size after preservation, whereas negative values indicate lower abundance, shrinkage, or reduced detectability. Where studies reported correction factors rather than direct percentage bias, those factors are shown as originally presented. “Apparent loss” denotes reduced detectability or altered classification rather than demonstrated disappearance of cells. “No significant change” indicates that the original study reported no statistically significant difference from the reference condition. For imaging-based studies, apparent abundance changes may reflect redistribution among size classes, aggregation, or altered detectability.

Abbreviations: HNAN, heterotrophic nanoflagellates; PNAN, phototrophic nanoflagellates; PPE, photosynthetic picoeukaryotes; QPS, quantitative protargol stain; DAPI, 4′,6-diamidino-2-phenylindole; FA, formaldehyde; GA, glutaraldehyde; F68, Pluronic F68; DMSO, dimethyl sulfoxide; HgCl2, mercuric chloride; LM, light microscopy; EFM, epifluorescence microscopy; ESD, equivalent spherical diameter; d, day(s); h, hour(s); wk, week(s); mL, millilitre; L, litre; µm, micrometre; µm³, cubic micrometre; °C, degrees Celsius. “Not measured” indicates that the corresponding variable was not quantified directly in the original study.

| **Study** | **Target group** | **Method / Fixative / Treatment** | **Abundance bias (%)** | **Reference (abundance)** | **Volume / size change (%)** | **Reference (volume)** | **Time scale / storage** | **Mechanism / interpretation** | **Detailed findings** |
| --- | --- | --- | --- | --- | --- | --- | --- | --- | --- |
| Bloem et al. 1986 | HNAN (Monas sp.) | 0.3% glutaraldehyde | 0% loss / stable | vs initial/live | Not reported | — | 4 weeks | Fixation | Stable preservation of Monas during fixation and storage; counts remained constant for several weeks. |
| Bloem et al. 1986 | HNAN (Monas sp.) | 5% formaldehyde | No loss during fixation; stable over weeks, but 11–20% lower at 4 weeks depending on count method | vs initial/live | Not reported | — | 4 weeks | Fixation | No loss of HNAN cells or flagella detected; stored samples remained constant for several weeks. |
| Bloem et al. 1986 | HNAN (Monas sp.) | Buffered (borax) 1% glutaraldehyde + 1% paraformaldehyde | 26-33% loss | vs initial/live | Not reported | — | 4 weeks | Fixation + chemistry | Buffered formulations had a strong negative effect on HNAN preservation. |
| Bloem et al. 1986 | HNAN (Monas sp.) | Borax-buffered formaldehyde | 100% loss in ~2 h | vs initial/live | Not reported | — | Immediate | Chemical damage | Total destruction under borax-buffered formaldehyde. |
| Bloem et al. 1986 | PNAN (Chlorella) | 0.3% glutaraldehyde | Fluorescence apparent 100% loss after 4 weeks/9% cell loss | vs fluorescence classification | Not reported | — | 4 weeks | Autofluorescence loss | Cells were present but became invisible as PNAN because fluorescence was lost. |
| Bloem et al. 1986 | HNAN (field) | 1% glutaraldehyde | No decline for at least 1 week | vs post-fixation baseline | Not reported | — | 1 week storage | Fixation | Field HNAN and total nanoflagellate concentrations remained stable for at least 1 week. |
| Bloem et al. 1986 | PNAN (field) | 1% glutaraldehyde | ~72% apparent loss after 1 day | vs initial fluorescence class | Not reported | — | 1 day storage | Fluorescence decay / misclassification | Rapid fluorescence decay caused PNAN to be misclassified as HNAN. |
| Bloem et al. 1986 | HNAN | Filtration (10 kPa) | 15-20% loss | vs untreated live sample | Not reported | — | Immediate | Mechanical stress | Vacuum filtration damaged cells even at relatively low pressure. |
| Bloem et al. 1986 | HNAN | Filtration (30 kPa) | 22-36% loss | vs untreated live sample | Not reported | — | Immediate | Mechanical stress | Higher vacuum caused severe losses. |
| Bloem et al. 1986 | HNAN | Centrifugation (500 g) | 18-34% loss | vs untreated live sample | Not reported | — | Immediate | Mechanical stress | Even relatively gentle centrifugation was harmful. |
| Bloem et al. 1986 | HNAN | Centrifugation (38,000 g) | 55-98% loss | vs untreated live sample | Not reported | — | Immediate | Cell destruction | Extreme losses at high centrifugal force. |
| Bloem et al. 1986 | PNAN/HNAN | Freezing slides at -30 C | ~0% loss | vs day-0 primulin-stained slide counts after fixation | Not reported | — | 16 weeks | Storage method | Primulin-stained slides could be stored frozen with excellent preservation. |
| Sime-Sime-Ngando & Groliere 1991 | Freshwater ciliates (all fixatives, overall mean) | All fixatives (mean) | -7.2% at 3 months | vs initial counts | Not measured | — | 3 months | Storage loss | Overall mean loss after 3 months was modest. |
| Sime-Ngando & Groliere 1991 | Freshwater ciliates (all fixatives, overall mean) | All fixatives (mean) | -16.4% at 6 months | vs initial counts | Not measured | — | 6 months | Storage loss | Overall mean loss increased by 6 months. |
| Sime-Ngando & Groliere 1991 | Freshwater ciliates (all fixatives, overall mean) | All fixatives (mean) | -30.7% at 9 months | vs initial counts | Not measured | — | 9 months | Storage loss | Overall mean loss after 9 months was substantial. |
| Sime-Ngando & Groliere 1991 | Freshwater ciliates | HgCl2 | -9.7% over 3-9 months | vs initial counts | Not measured | — | 9 months | Fixative effect | Best preservation among the tested fixatives. |
| Sime-Ngando & Groliere 1991 | Freshwater ciliates | Lugol | -57.9% at 9 months | vs initial counts after fixation | Not measured | — | 9 months | Fixative effect | Worst preservative by far for long-term ciliate storage. |
| Sime-Ngando & Groliere 1991 | Freshwater ciliates | Formaldehyde, glutaraldehyde, Champy-Da Fano | ~10% at 6 months; ~26% at 9 months | vs initial counts after fixation | Not measured | — | 6-9 months | Fixative effect | The three non-HgCl2, non-Lugol fixatives reduced ciliate numbers by around 10% at 6 months and 26% at 9 months. |
| Sime-Ngando & Groliere 1991 | Freshwater ciliates | Filtration or centrifugation before fixation | 1.3-fold greater sensitivity to storage losses | vs unconcentrated samples | Not measured | — | Up to 9 months | Handling stress | Pre-stressed ciliates were more sensitive to conservation than unconcentrated samples. |
| Sime-Ngando & Groliere 1991 | Scuticociliates | All fixatives | More affected than oligotrichs | relative among taxa | Not measured | — | Up to 9 months | Taxon-specific sensitivity | Scuticociliates were more sensitive to storage than oligotrichs and some other taxa. |
| Sime-Ngando & Groliere 1991 | Oligotrichs | All fixatives | Less affected than scuticociliates | relative among taxa | Not measured | — | Up to 9 months | Taxon-specific sensitivity | Oligotrichs appeared relatively resistant. |
| James 1991 | Ciliates <50 um | 1% Lugol vs 2% glutaraldehyde | +31.1% higher with Lugol (231.6 vs 176.7 cells L^-1^) | Lugol relative to glutaraldehyde | Not reported | — | Preservation comparison | Fixation distortion / detectability | Small ciliates were significantly more abundant in Lugol-preserved samples than in glutaraldehyde. |
| James 1991 | Ciliates <50 um | 1% Lugol vs 2% formalin | +65.0% higher with Lugol (231.6 vs 140.4 cells L^-1^) | Lugol relative to formalin | Not reported | — | Preservation comparison | Fixation distortion / detectability | Lugol strongly outperformed formalin for ciliates <50 um. |
| James 1991 | Ciliates <50 um | 1% Lugol vs HgCl2 + bromophenol blue | +13.5% higher with Lugol (231.6 vs 204.0 cells L^-1^) | Lugol relative to HgCl2 | Not reported | — | Preservation comparison | Fixation distortion / detectability | Lugol and HgCl2 gave the highest small-ciliate counts. |
| James 1991 | Rhizopodea | Lugol vs glutaraldehyde/formalin/HgCl2 | Present in Lugol (Lugol much higher (86.9 vs 4.0, 1.3, and 4.0 cells L⁻¹) but near-absent or absent in aldehydes | counts by fixative | Not reported | — | Preservation comparison | Fragility | Rhizopodea were common only in Lugol-preserved settled samples; aldehydes gave near-total loss. |
| James 1991 | Heliozoa | Lugol vs glutaraldehyde | +152.4% higher with Lugol (91.1 vs 36.1 cells L^-1^) | Lugol relative to glutaraldehyde | Not reported | — | Preservation comparison | Structural preservation | Heliozoa were much better preserved in Lugol than in glutaraldehyde. |
| James 1991 | Heliozoa | Lugol vs formalin | +224.2% higher with Lugol (91.1 vs 28.1 cells L^-1^) | Lugol relative to formalin | Not reported | — | Preservation comparison | Structural preservation | Heliozoa were strongly underestimated in formalin. |
| James 1991 | Small ciliates (<50 um) | Mesh screening / pumping / net haul methods | Large underestimation | relative to settled Van Dorn bottle samples | Not reported | — | Sampling comparison | Size escape + mechanical stress | Sampling method strongly affected enumeration of small protozoa. |
| James 1991 | Ciliates <50 um | 20 um mesh gravity filtration | 100% pass-through | fractionation test | Not reported | — | Immediate | Size mismatch | All ciliates <50 um passed through a 20 um mesh. |
| James 1991 | Ciliates <50 um | 10 um mesh gravity filtration | 34% pass-through | fractionation test | Not reported | — | Immediate | Size mismatch | Even 10 um mesh still lost about one-third of the small ciliate fraction. |
| Stoecker et al. 1994 | Strombidium capitatum (culture) | 2% formaldehyde vs 10% acid Lugol | -36% (193 vs 300 cells) | vs Lugol | Formaldehyde volume +145% relative to Lugol; Lugol approx. -59% relative to formaldehyde | vs Lugol | Culture experiment | Abundance-size tradeoff | Formaldehyde preserved larger cell size but lost many cells relative to Lugol. |
| Stoecker et al. 1994 | Strobilidium spiralis (culture) | 2% formaldehyde vs 10% acid Lugol | -17% (138 vs 167 cells) | vs Lugol | Formaldehyde volume +69% relative to Lugol; Lugol approx. -41% relative to formaldehyde | vs Lugol | Culture experiment | Abundance-size tradeoff | Moderate abundance loss in formaldehyde but much less shrinkage than in Lugol. |
| Stoecker et al. 1994 | Favella sp. (tintinnid) | 2% formaldehyde vs 10% acid Lugol | -11% (246 vs 277 cells) | vs Lugol | Formaldehyde volume +78% relative to Lugol; Lugol approx. -44% relative to formaldehyde | vs Lugol | Culture experiment | Abundance-size tradeoff | Tintinnids showed relatively small abundance loss but large Lugol shrinkage. |
| Leakey et al. 1994 | Total ciliates | 1% formaldehyde vs 0.4% acid Lugol | -36% | vs Lugol | +21% volume relative to Lugol | vs Lugol | Field comparison | Abundance-size tradeoff | Formaldehyde recovered fewer cells than Lugol but yielded larger measured cells. |
| Leakey et al. 1994 | Total ciliates | 1% glutaraldehyde vs 0.4% Lugol | -31% | vs Lugol | +61.1% volume relative to Lugol | vs Lugol | Field comparison | Abundance-size tradeoff | Glutaraldehyde showed slightly better abundance recovery than formaldehyde but much larger volumes than Lugol. |
| Leakey et al. 1994 | Aloricate ciliates | 1% formaldehyde vs 0.4% Lugol | -48% | vs Lugol | +5% volume relative to Lugol | vs Lugol | Field comparison | Abundance-size tradeoff | Strong cell-number underestimation with only small volume difference relative to Lugol. |
| Leakey et al. 1994 | Aloricate ciliates | 1% glutaraldehyde vs 0.4% Lugol | -45% | vs Lugol | +12.2% volume relative to Lugol | vs Lugol | Field comparison | Abundance-size tradeoff | Glutaraldehyde also underestimated aloricate abundance strongly. |
| Leakey et al. 1994 | Tintinnids | 1% formaldehyde vs 0.4% Lugol | -1.4% | vs Lugol | +94.0% volume relative to Lugol | vs Lugol | Field comparison | Abundance-size tradeoff | Tintinnid counts changed little between fixatives, but Lugol gave much smaller measured volumes. |
| Leakey et al. 1994 | Tintinnids | 1% glutaraldehyde vs 0.4% Lugol | +1.6% | vs Lugol | +24.8% volume relative to Lugol | vs Lugol | Field comparison | Abundance-size tradeoff | Tintinnids were resistant in abundance terms but still showed fixative-dependent volume differences. |
| Pfister et al. 1999 | Total ciliates (field, lake) | QPS (Bouin + protargol) vs live | -1% (29.5 vs 29.9 cells mL-1; not significant) | vs live | Strong shrinkage (taxon-specific) | vs live | Method comparison | Method-specific bias | Total ciliate abundance was accurately recovered overall despite taxon-specific deviations. |
| Pfister et al. 1999 | Naked oligotrichs | QPS vs live | -48 to -74% | vs live | Severe shrinkage | vs live | Method comparison | Fragility + misidentification | Fragile naked oligotrichs were strongly underestimated in QPS. |
| Pfister et al. 1999 | Loricate oligotrichs | QPS vs live | -8 to +33% | vs live | Moderate shrinkage (~25%) | vs live | Method comparison | Improved detectability | Loricate oligotrichs could be overcounted relative to live counts. |
| Pfister et al. 1999 | Prostomatids | QPS vs live | -16 to +67% (depth-dependent) | vs live | Not explicitly quantified | — | Method comparison | Taxonomic resolution effect | Increase likely reflects better recognition in QPS rather than pure preservation gain. |
| Pfister et al. 1999 | Hymenostomatids | QPS vs live | +200 to +600% | vs live | Strong shrinkage (<20% of live volume) | vs live | Method comparison | Taxonomic resolution effect | Positive bias may partly reflect improved detectability or taxonomic resolution in QPS preparations. |
| Pfister et al. 1999 | Peritrichs | QPS vs live | -37 to +300% | vs live | Not specified | — | Method comparison | High variability | Response depended strongly on sample and depth. |
| Pfister et al. 1999 | Measured flagellate species (field) | QPS vs live | -70% (QPS about 30% of live) | vs live | -30 to -63% | vs live | Method comparison | Misidentification + cell loss | Flagellates were severely underestimated by QPS. |
| Pfister et al. 1999 | Ciliates (cultures) | QPS vs live | Halteria -25%; Cyclidium -36%; Cinetochilum -66% | vs live | Strong shrinkage | vs live | Culture comparison | Fragility gradient | Underestimation increased with fragility. |
| Sonntag et al. 2000 | Freshwater flagellates (multiple taxa) | Formaldehyde + DAPI vs live | -22% to +15% | vs live | -81% to +8% | vs live | Method comparison | Species-specific response | Fo/DAPI ranged from 78% to 115% of live abundance; volume from 19% to 108% of live. |
| Sonntag et al. 2000 | Freshwater flagellates (multiple taxa) | QPS (Bouin + stain) vs live | -37% to +4% | vs live | -30% to +37% | vs live | Method comparison | Species-specific response | QPS ranged from 63% to 104% of live abundance; volume from 70% to 137% of live. |
| Sonntag et al. 2000 | Flagellate taxa examined | All methods | Up to -56% | vs live | -81% to +37% | vs live | Method comparison | Taxon-specific variability | No universal correction factor is possible across taxa and methods. |
| Sonntag et al. 2000 | Small freshwater flagellates (<5 um) | Live counting | Severe underestimation | methodological detection limit | Not reported | — | Immediate | Detection bias | Bias here reflects live-counting limits rather than fixation only. |
| Menden-Deuer et al. 2001 | Thecate dinoflagellates | Lugol + glutaraldehyde (combined) | Not measured | — | 0 to +30% (mean about +16%) | vs live | Method comparison | Thecate swelling | Only group showing consistent swelling. |
| Menden-Deuer et al. 2001 | Athecate dinoflagellates | Lugol + glutaraldehyde (combined) | Not measured | — | -23% to +5% (mean about -9%) | vs live | Method comparison | Mixed but generally shrinking | Moderate shrinkage dominated in athecate taxa. |
| Menden-Deuer et al. 2001 | Diatoms (8 spp.) | LM, Lugol + glutaraldehyde combined | Not measured | — | -33% to +28% | vs live | Method comparison | Species-specific response | Diatoms were not immune to preservation artifacts. |
| Menden-Deuer et al. 2001 | Dinoflagellates (all) | 1% glutaraldehyde + filtration + EFM | Not measured | — | -69% to +5% (mean about -32%) | vs live | Method comparison | Method-induced shrinkage | Filtration introduced systematic shrinkage. |
| Menden-Deuer et al. 2001 | Dinoflagellates and diatoms | Lugol / glutaraldehyde time series | Not measured | — | -10% to +46% over time | vs live | Storage time series | Non-linear time effect | Non-linear, species-specific change over time; from modest initial shrinkage to +46% swelling in dinoflagellates, and >50% shrinkage in some diatoms during long storage |
| Chaput & Carrias 2002 | Flagellates (Carteria sp., Cryptomonas sp., Bodo saltans) | Formaldehyde (2%) | Not measured | — | −59.4 to ~0 | vs live | Method comparison | Species-specific response | No significant volume change for Carteria sp. or Cryptomonas sp.; strong shrinkage in Bodo saltans (−59.4%). |
| Chaput & Carrias 2002 | Flagellates (Carteria sp., Cryptomonas sp., Bodo saltans) | Glutaraldehyde (1%) | Not measured | — | −64.2 to ~0 | vs live | Method comparison | Species-specific response | No significant volume change for Carteria sp.; little or no effect on Cryptomonas sp.; very strong shrinkage in Bodo saltans (−64.2%). |
| Chaput & Carrias 2002 | Flagellates (Carteria sp., Cryptomonas sp., Bodo saltans) | Lugol’s iodine (1%) | Not measured | — | −53.3 to −7.9 | vs live | Method comparison | Species-specific response | All three flagellates decreased in volume; approximate responses were Carteria sp. −25.3%, Cryptomonas sp. −7.9%, and Bodo saltans −53.3%. |
| Chaput & Carrias 2002 | Flagellates (Carteria sp., Cryptomonas sp., Bodo saltans) | Mercuric chloride (2.5%) | Not measured | — | −38.1 to +34.5 | vs live | Method comparison | Species-specific response | Highest interspecific variability among flagellates; Bodo saltans shrank (−38.1%), Cryptomonas sp. swelled (+34.5%), and Carteria sp. showed no significant change. |
| Chaput & Carrias 2002 | Ciliates (Uronema sp., Cyclidium glaucoma, Paramecium caudatum) | Formaldehyde (2%) | Not measured | — | ~0 to +53.8 | vs live | Method comparison | Species-specific response | No significant volume effect on Uronema sp. or Paramecium caudatum; strong swelling in Cyclidium glaucoma (+53.8%). |
| Chaput & Carrias 2002 | Ciliates (Uronema sp., Cyclidium glaucoma, Paramecium caudatum) | Glutaraldehyde (1%) | Not measured | — | −38.5 to +44.0 | vs live | Method comparison | Species-specific response | Opposite responses within ciliates; Uronema sp. shrank (−38.5%), Cyclidium glaucoma swelled strongly (~+44%), and Paramecium caudatum showed no significant change. |
| Chaput & Carrias 2002 | Ciliates (Uronema sp., Cyclidium glaucoma, Paramecium caudatum) | Lugol’s iodine (1%) | Not measured | — | −35.4 to +42.2 | vs live | Method comparison | Species-specific response | Mixed response; Uronema sp. shrank (−35.4%), Paramecium caudatum shrank (−32.6%), and Cyclidium glaucoma swelled (+42.2%). |
| Chaput & Carrias 2002 | Ciliates (Uronema sp., Cyclidium glaucoma, Paramecium caudatum) | Mercuric chloride (2.5%) | Not measured | — | +14.8 to +30.0 | vs live | Method comparison | Species-specific response | All ciliates tended to swell; Cyclidium glaucoma increased by +14.8%, Paramecium caudatum by +28.0%, and Uronema sp. by +30.0%. |
| Broglio et al. 2004 | Strombidium sulcatum | 10% acid Lugol | -30% | vs live | Not reported | — | Preservation correction used by authors | Species-specific correction | The paper reports a 30% reduction in cell counts after preservation in 10% acidic Lugol and applies that correction; this should not be generalized to all naked ciliates. |
| Modigh & Castaldo 2005 | Total ciliates | 2% borate formaldehyde vs 1% Lugol | Lugol counts were on average 33% higher than formaldehyde counts | vs Lugol | Not reported directly here | — | Field comparison | Abundance underestimation | Counts in Lugol were on average 33% higher than in formaldehyde. |
| Modigh & Castaldo 2005 | Naked ciliates <18 um ESD | 2% borate formaldehyde vs 1% Lugol | Correction factor 1.25 (formal to Lugol equivalent) | vs Lugol | Not reported separately | — | Field comparison | Size-dependent correction | Use 1.25 to correct formaldehyde counts for the smallest naked ciliates. |
| Modigh & Castaldo 2005 | Naked ciliates 18-30 um ESD | 2% borate formaldehyde vs 1% Lugol | Correction factor 1.44 | vs Lugol | Not reported separately | — | Field comparison | Size-dependent correction | Intermediate naked ciliates required stronger correction than the smallest size class. |
| Modigh & Castaldo 2005 | Naked ciliates >30 um ESD | 2% borate formaldehyde vs 1% Lugol | Correction factor 1.50 | vs Lugol | Not reported separately | — | Field comparison | Size-dependent correction | Largest naked ciliates were most underestimated in formaldehyde. |
| Modigh & Castaldo 2005 | Mesodinium rubrum | 2% borate formaldehyde vs 1% Lugol | Correction factor 1.60 | vs Lugol | Not reported separately | — | Field comparison | Taxon-specific correction | Mesodinium rubrum required a stronger correction than most other groups. |
| Modigh & Castaldo 2005 | Mixotrophic ciliates | 2% borate formaldehyde vs 1% Lugol | Correction factor 1.17 | vs Lugol | Not reported separately | — | Field comparison | Taxon-specific correction | Mixotrophs were less underestimated than most naked ciliates. |
| Modigh & Castaldo 2005 | Tintinnids | 2% borate formaldehyde vs 1% Lugol | Correction factor 0.99 | vs Lugol | Not reported separately | — | Field comparison | Taxon-specific correction | Tintinnid abundance was essentially unaffected by the fixative choice. |
| Modigh & Castaldo 2005 | Naked ciliate biomass | 2% borate formaldehyde vs 1% Lugol | Not reported as simple % loss | — | Biomass correction factor 1.81 | formaldehyde to Lugol-equivalent biomass | Field comparison | Biomass underestimation | Naked ciliate biomass was more strongly underestimated than abundance. |
| Zarauz & Irigoien 2008 | Natural nano-microplankton (8-200 um ESD) | Lugol's 1% | 8-20 um: -70%; 20-40 um: +25%; 40-200 um: -60% | vs live samples | Assumed shrinkage correction: fixed volume × 1.33 to estimate live volume (~25% smaller when fixed) | vs live samples | Method comparison | Aggregation + shrinkage + damage | Strong size-dependent redistribution; fragile taxa, especially ciliates, were strongly biased. |
| Zarauz & Irigoien 2008 | Natural nano-microplankton (8-200 um ESD) | Lugol's 5% | 8-20 um: -69%; 20-40 um: +276%; 40-200 um: +46% | vs live samples | ~ -25% assumed / not re-estimated | assumed by authors | Method comparison | Aggregation + redistribution | Higher Lugol concentration amplified redistribution toward intermediate and larger apparent classes. |
| Zarauz & Irigoien 2008 | Natural nano-microplankton (8-200 um ESD) | 5% Lugol vs 1% Lugol | 8-20 um: -21%; 20-40 um: +18%; 40-200 um: +39% | 5% relative to 1% Lugol | Not quantified | — | Method comparison | Concentration effect | Increasing Lugol concentration changed size structure rather than causing a uniform loss. |
| Zarauz & Irigoien 2008 | Fragile taxa (including ciliates) | Lugol's (general) | Not quantified | qualitative vs live | Shrinkage and structural damage (qualitative) | qualitative vs live | Method comparison | Fragility / staining | Ciliates lost cilia, shrank, and stained strongly, reducing detectability. |
| Jakobsen & Carstensen 2011 | Mixed protist community | Lugol (4%) vs live | Decrease across all size classes; no single universal % | vs live community | -60.4% total biovolume (13.34 to 5.29) | vs live community | Community comparison | Fragmentation + aggregation + loss of large cells | Lugol strongly underestimated total biomass and shifted the size spectrum. |
| Jakobsen & Carstensen 2011 | Size-structured community | Lugol (4%) vs live | Relative decrease in counts across ESD classes | vs live spectrum | Systematic shift toward smaller ESD; broadening of distribution | vs live spectrum | Community comparison | Size-structure distortion | Fragmentation and aggregation redistributed particles among bins. |
| Jakobsen & Carstensen 2011 | Large protists incl. ciliates | Lugol (4%) | Strong qualitative loss | vs live observations | Large indirect contribution to total biomass decline | vs live | Community comparison | Disappearance of large cells | Loss of large particles drove much of the biomass underestimation. |
| Yang et al. 2017 | Prorocentrum micans | Lugol (0.6-0.8%) | Not reported | — | 0% long-term; transient shrinkage to about -6.16% | vs live | Short- and long-term | Species-specific response | Initial shrinkage was followed by recovery to live-equivalent size after about 1 month. |
| Yang et al. 2017 | Prorocentrum micans | Formalin (5%) | Not reported | — | -1.91% at 2 h; about 0% long-term | vs live | Short- and long-term | Species-specific response | Only slight short-term shrinkage; no long-term bias. |
| Yang et al. 2017 | Scrippsiella trochoidea | Lugol (0.6-0.8%) | Not reported | — | +8.06% long-term; +1.32% short-term | vs live | Short- and long-term | Species-specific response | This thecate species swelled slightly under Lugol. |
| Yang et al. 2017 | Scrippsiella trochoidea | Formalin (5%) | Not reported | — | +20.97% long-term; up to +23.77% short-term | vs live | Short- and long-term | Species-specific response | Formalin caused strong swelling and biomass overestimation if uncorrected. |
| Yang et al. 2017 | Noctiluca scintillans | Lugol (0.6-0.8%) | 14-27% breakage | damage rate within fixative | -27.6% long-term; about -3.3% ESD-based in table | vs live | Short- and long-term | Shrinkage + rupture | Moderate shrinkage but substantial cell rupture. |
| Yang et al. 2017 | Noctiluca scintillans | Lugol (2%) | Breakage up to 73% | damage rate within fixative | -69.1% long-term; -50.6% at 2 h | vs live | Short- and long-term | Concentration-dependent destruction | High-concentration Lugol produced extreme shrinkage and damage. |
| Yang et al. 2017 | Noctiluca scintillans | Formalin (5%) | 1.52% at 2 h; ~30–36.8% by the end of the time series | damage rate within fixative | −39.36% long-term volume; −12.26% long-term ESD | vs live | Short- and long-term | Shrinkage + damage | Formalin still produced large bias, though less concentration-sensitive than Lugol. |
| Yang et al. 2017 | Noctiluca scintillans | Formalin (10%) | Similar breakage to 5% | 5% formalin as reference | No significant additional shrinkage vs 5% | within-formalin comparison | Short- and long-term | Weak concentration dependence | Formalin effects were less concentration-dependent than Lugol effects. |
| Liu et al. 2022 | PPE (lakes) | Formaldehyde (2%), frozen at -80º C | Abundance close to fresh, but composition severely distorted | vs fresh | Not reported | — | 30-180 days | Fluorescence preserved but composition distorted | Aldehydes preserved fluorescence sufficiently for abundance analysis, but PPE proportion dropped from about 90% in fresh samples to <10%. |
| Liu et al. 2022 | PPE (lakes) | Glutaraldehyde (0.25%), frozen at -80º C | Abundance close to fresh, but composition severely distorted | vs fresh | Not reported | — | 30-180 days | Fluorescence preserved but composition distorted | Glutaraldehyde behaved similarly to formaldehyde for abundance analysis but severely altered apparent composition. |
| Liu et al. 2022 | PPE (lakes) | FA + F68 or GA + F68, frozen at -80º C | PPE proportion remained close to fresh samples, at ~86%, 84%, and 53% after 6 months depending on lake. | vs fresh | Not reported | — | 30-180 days | Cryoprotection / compositional bias | Adding F68 to aldehydes improved handling but did not restore true community structure. |
| Liu et al. 2022 | PPE (lakes) | DMSO (with or without F68), frozen at -80º C | Strong decrease / unreliable | vs fresh | Not reported | — | Storage | Fluorescence loss + structural damage | DMSO damaged fluorescence and cell structure, making counting/sorting unreliable. |
| Liu et al. 2022 | PPE (lakes) | F68 (0.01%), frozen at -80º C | Stable abundance | vs fresh | Not reported | — | Up to 180 days | Best overall protocol | PPE proportion remained close to fresh samples, at ~86%, 84%, and 53% after 6 months depending on lake. |
| Nogueira et al. 2023 | Total microphytoplankton (>20 um) | Acid Lugol (~0.2%), dark, 15-20º C | -39% at 90 d; -51% at 365 d | vs initial | Not reported | — | 90-365 days | Progressive storage decline | Progressive decline with stabilization after about 90 days. |
| Nogueira et al. 2023 | Plastidic dinoflagellates | Acid Lugol (~0.2%), dark, 15-20º C | -36% at 90 d; -44% at 365 d | vs initial | Not reported | — | 90-365 days | Progressive storage decline | Pattern resembled total microphytoplankton decline. |
| Nogueira et al. 2023 | Diatoms | Acid Lugol (~0.2%), dark, 15-20º C | -62% at 365 d | vs initial | Not reported | — | 365 days | Continuous decline | Diatoms continued declining without stabilization. |
| Nogueira et al. 2023 | Cyanobacteria | 2% glutaraldehyde refrigerated at 4º C | Stable over 0-14 d | vs initial | Not reported | — | 0-14 days | Good short-term storage | No significant refrigerated storage effect. |
| Nogueira et al. 2023 | Cryptophytes | 2% glutaraldehyde refrigerated at 4º C | Stable over 0-14 d | vs initial | Not reported | — | 0-14 days | Good short-term storage | Stable abundances over 14 days. |
| Nogueira et al. 2023 | Eukaryotic picophytoplankton | 2% glutaraldehyde refrigerated at 4º C | Stable <=4 d; -50 to -60% by about 14 d | vs initial | Not reported | — | 0-14 days | Rapid late decline | Sharp decline after day 4. |
| Nogueira et al. 2023 | Plastidic nanoflagellates | 2% glutaraldehyde refrigerated at 4 ºC | Stable <=4 d; -45 to -50% by about 14 d | vs initial | Not reported | — | 0-14 days | Rapid late decline | Similar decline to picoeukaryotes. |
| Nogueira et al. 2023 | Cyanobacteria | 2% glutaraldehyde, microscopy slides stored at −20 °C | Relatively stable, with only modest decline over 1 year; storage effect statistically significant in Table I | vs initial | Not reported | — | 1 year | Good frozen-slide stability | No long-term decline detected. |
| Nogueira et al. 2023 | Cryptophytes | 2% glutaraldehyde, microscopy slides stored at −20 °C | Stable to 1 year | vs initial | Not reported | — | 1 year | Good frozen-slide stability | No significant long-term change. |
| Nogueira et al. 2023 | Eukaryotic picophytoplankton | 2% glutaraldehyde, microscopy slides stored at −20 °C | -27% at 7 d; -70 to -80% by 365 d | vs initial | Not reported | — | 7-365 days | Rapid early loss + exponential decline | Slides should be prepared immediately before observation when possible. |
| Nogueira et al. 2023 | Plastidic nanoflagellates | 2% glutaraldehyde, microscopy slides stored at −20 °C | -43% at 7 d; -60 to -75% by 365 d | vs initial | Not reported | — | 7-365 days | Rapid decline then stabilization | Strong long-term decline; stabilizes after about 90 d. |
